# Supplementary material for: Does Zipf’s law of abbreviation shape birdsong?
Source: PLoS Comput Biol. 2025 Aug 13;21(8):e1013228. doi: 10.1371/journal.pcbi.1013228 (PMC12349147; doi:10.1371/journal.pcbi.1013228)
Supplement: S1 Table — (PDF) [file pcbi.1013228.s001.pdf]

species	records (birds) studied	total phrase types	phrases per record	phrase types per record	Shannon diversity	concordance (population)	mean concordance (individual)	maximum significant concordance
California thrasher	89	748	145.7	14.4	2.20	**-0.076 (p = 0.001)**	0.029 (p = 0.890)	-0.039
	7	181	411.6	57.4	3.62	-0.070 (p = 0.086)	-0.058 (p = 0.115)	-0.080
redthroat	7	56	175.6	14.9	2.15	-0.044 (p = 0.319)	-0.024 (p = 0.394)	-0.144
black-headed grosbeak	83	451	153.4	27.5	2.92	-0.004 (p = 0.451)	-0.045 (p = 0.068)	-0.050
	16	107	109.4	25.9	2.93	**0.167 (p = 0.993)**	-0.039 (p = 0.282)	-0.111
sage thrasher	2	147	234.0	89.5	4.13	-0.043 (p = 0.241)	-0.038 (p = 0.276)	-0.103
Cassin’s vireo	13	68	87.7	26.5	2.98	**0.167 (p = 0.975)**	-0.065 (p = 0.196)	-0.124
	296	134	119.1	21.5	2.54	-0.031 (p = 0.296)	-0.028 (p = 0.192)	-0.053
	41	114	94.6	26.6	2.85	0.091 (p = 0.922)	-0.028 (p = 0.250)	-0.067
western tanager	3	56	128.7	22.7	2.30	**-0.176 (p = 0.034)**	-0.152 (p = 0.057)	-0.158
grey shrike-thrush	4	9	13.3	2.8	0.76	-0.032 (p = 0.455)	-0.166 (p = 0.313)	-0.550

Concordances between note duration and frequency of use in the songs of 11 populations of 7 bird species archived on Bird-DB when note durations are represented by medians rather than means. P-values less than 0.05 indicate patterns strongly consistent with ZLA (bold red), and p-values greater than 0.95 indicate patterns strongly contrary to ZLA (bold blue).
