# Supplementary material for: Does Zipf’s law of abbreviation shape birdsong?
Source: PLoS Comput Biol. 2025 Aug 13;21(8):e1013228. doi: 10.1371/journal.pcbi.1013228 (PMC12349147; doi:10.1371/journal.pcbi.1013228)
Supplement: S1 Appendix — (PDF) [file pcbi.1013228.s002.pdf]

S1 Appendix

In this appendix, we illustrate the relationship between the standard deviations of the durations of individual note or phrase types as produced by different birds and the expected durations of those same note or phrase types in the population. We use the two populations from Bird-DB with the largest number of recorded phrase types as examples. One population is represented by 89 California thrashers that produced a total of 748 phrase types, and the other is represented by 83 black-headed grosbeaks that produced a total of 451 phrase types.

In Figure A, panels (a,b) show the standard deviations of phrase types as produced by different birds plotted as functions of the population mean durations for those same phrase types in (a) California thrashers and (b) black-headed grosbeaks. The standard deviations increase with the means. The red lines show the log-linear relationships between the standard deviations and the means. The slopes and p-values for those relationships are reported in Table A.

Panels (c,d) show the distributions of phrase type durations for (c) California thrashers and (d) black-headed grosbeaks. The distributions are right-skewed, and thus the means (vertical red lines) are greater than the medians (vertical blue lines). Table A reports the mean, median, and percentile rank of the mean for each distribution.

Panels (e-h) are analogous to panels (a-d), respectively, but for the log-transformed phrase durations. There are no lines of best fit in (e,f) because there is no significant relationship between the standard deviations and the means of log-transformed phrase type durations (Table A). The distributions of log-transformed phrase type durations are

more symmetrical than the distributions of raw phrase type durations, and thus the means are more similar to the medians (f,g; Table A).

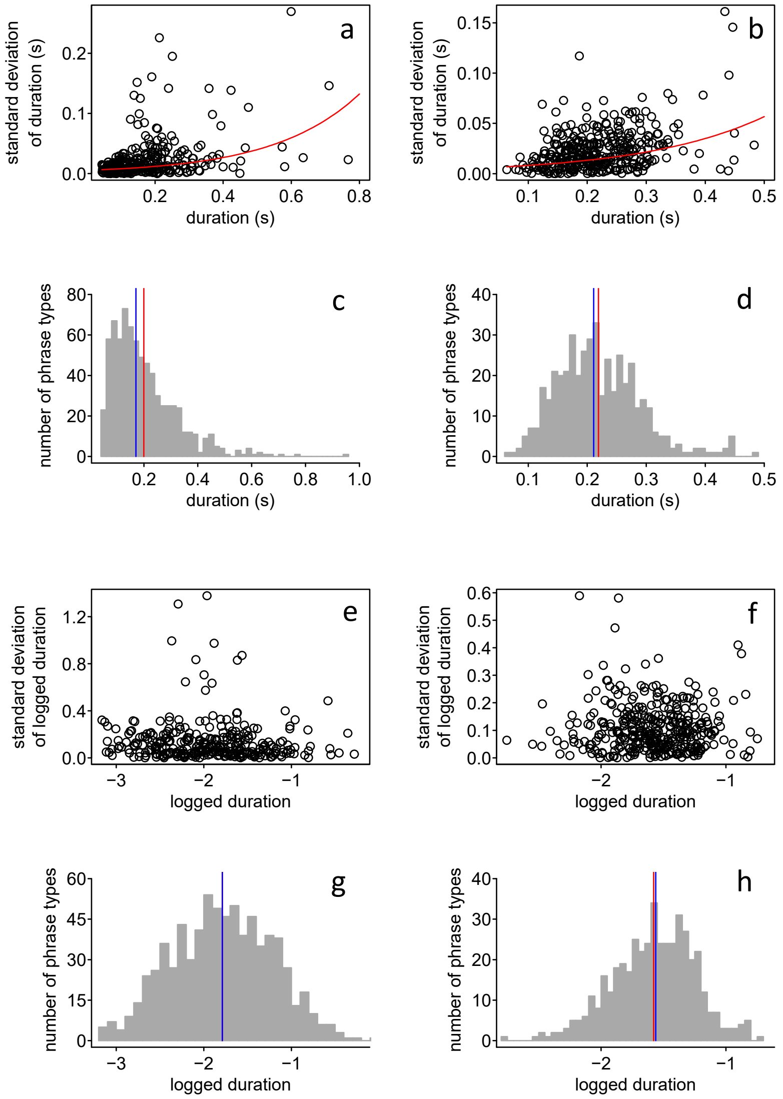

Figure A. The standard deviations of phrase type durations as produced by different birds plotted as functions of the means for (a) California thrashers and (b) black-headed grosbeaks. Red lines of best fit show the log-linear relationships. (c,d) The distributions of population mean durations for the phrase types illustrated in (a,b), respectively. Blue vertical lines show medians and red vertical lines show means. Panels (e-h) are analogous to (a-d), respectively, but for log-transformed phrase durations.

population	measure	mean of measure	median of measure	percentile rank of mean	slope of the log-transformed standard deviation on the mean	p-value for log-linear relationship
89 California thrashers	raw duration	0.200 s	0.170 s	0.600	4.00 s^{-1}	<0.001
89 California thrashers	log-transformed duration	-1.79	-1.79	0.501	-0.201	0.0974
83 black-headed grosbeaks	raw duration	0.219 s	0.211 s	0.550	4.84 s^{-1}	<0.001
83 black-headed grosbeaks	log-transformed duration	-1.58	-1.56	0.475	0.0002	0.999

Table A. Properties of the relationships between the standard deviations of phrase type durations as produced by different birds and the population means for those same phrase type durations.
