# Supplementary material for: Does Zipf’s law of abbreviation shape birdsong?
Source: PLoS Comput Biol. 2025 Aug 13;21(8):e1013228. doi: 10.1371/journal.pcbi.1013228 (PMC12349147; doi:10.1371/journal.pcbi.1013228)
Supplement: S2 Appendix — (PDF) [file pcbi.1013228.s003.pdf]

**S2 Appendix**

We studied simulated birdsong to understand how the misclassification of notes affects our inferences about ZLA. We simulated populations in which birdsong either adhered to or did not adhere to ZLA, and we sampled from these populations with and without note classification errors. We studied these samples using ZLAvian as described in the body of the paper. We recorded the false positive rate (ie, the proportion of populations that did not adhere to ZLA but in which we incorrectly inferred ZLA) and the power (ie, the proportion of populations that adhered to ZLA and in which we correctly inferred ZLA) of our test when notes were correctly or incorrectly classified.

Each population we simulated had a repertoire of *N* note types. In nature, the relative frequencies with which note types are used by bird populations often declines with the rank order frequencies of use following a Zipf-Mandelbrot law [42]. Thus, the $r^{\text{th}}$ most frequently used note type has a relative frequency of use

$$f(r) \approx \frac{c}{(r+\beta)^{\alpha}}$$

where $\alpha$ and $\beta$ are parameters specific to the population and $c = 1/\sum_i (i+\beta)^{-\alpha}$ is a normalizing constant that ensures the relative frequencies of use sum to 1. We obtained the expected frequencies of use for the *N* note types in our simulated repertoires by setting $\alpha = 1.20$ and $\beta = 5.69$. Youngblood [42] estimated these parameter values in a well-studied population of house finches. To test whether our inferences about the effects of classification errors depend on the note frequency distribution, we confirmed our qualitative results when relative frequencies of use declined exponentially with rank order frequencies of use.

In many real bird populations, the songs of individual birds are not independent because birds learn their songs from others. To simulate this, we allowed each population to be composed of *L* song lineages (sensu [47]) in which each bird learned his song, possibly with errors, from his father. Each lineage included one founder, all his sons, all his paternal grandsons, and so on. For simplicity, we assumed that every lineage included *g* generations after the founder, and that each bird in every generation except the last had *h* sons. Thus, *L*, *g*, and *h* controlled the size of the simulated populations. We assumed that each bird in each simulated population produced a song comprising *n* notes. To simulate the song of each founder, we sampled *n* notes with replacement from the *N* note types in the population repertoire, with each note type chosen with a probability proportional to its expected frequency of use. Thus, each founder's song might include multiple instances of some note types and no instances of others. To simulate the song of each son, we selected each of the *n* notes in his song independently as follows: with probability *f* we selected a note from his father's song, with each individual note (not note type) in the father's song equally likely to be chosen, and we added a note of that type to the son's song; and with probability $(1 - f)$ we selected a note type from the population repertoire, where each note type was chosen with a probability proportional to its expected frequency of use. Thus, the songs of sons were similar but not identical to the songs of their fathers, and the songs of birds from the same lineage were more similar to each other than to the songs of birds from different lineages.

To simulate populations in which birdsong adheres to ZLA, we assigned each note type a duration that corresponded to the rank order of its expected frequency of use in the population. Thus, we assigned the shortest duration to the note type with the greatest

expected frequency of use, the second shortest duration to the note type with the second greatest expected frequency of use, and so on. To simulate populations in which birdsong does not adhere to ZLA, we assigned each note class a duration independent of its frequency of use.

We simulated three types of classification errors that might occur. For each type of classification error, we simulated $m$ misclassifications per population. To simulate the merger of note types, we assumed that note types were most likely to be erroneously merged if their durations were similar. We sampled, without replacement, $m$ note types from among the 2nd to $N$th longest note types in the population repertoire, and we merged these note types with the first longer note type that was not among the $m$ note types we sampled. For example, if our $m$ note types included the 3rd longest note type but not the 4th longest note type then we merged the 2nd and 3rd longest note types, and if our $m$ note types included the 3rd and 4th longest note types then we merged the 2nd through 4th longest note types. After mergers, the population repertoire included $(N - m)$ note types. Because we merged notes with consecutive rank order durations, we could assign rank order durations to note types after mergers without ambiguity. If note classes are erroneously split, the splitting might occur within birds or it might occur among birds. For example, in a real system we might erroneously split within birds if individual birds produce slightly different versions of a note type at different points in their songs, and we might erroneously split among birds if different birds produce slightly different versions of the same note type. For each type of erroneous splitting, we sampled, without replacement, $m$ note types from the population repertoire that would be split. To simulate splitting within birds, we considered each individual note in the set of $m$ note types that we sampled, and with

probability 0.5 we assigned that note to a new note type. To simulate splitting among birds, for each of our $m$ note types we considered each bird in the population. If the bird used that note type, then with probability 0.5 we assigned all of that bird's instances of that note type to a new type. In either case, if note type $i$ was among the $m$ types we split, all notes that were originally of type $i$ but were split from that type were assigned to the same new type $i'$, so after splitting the population repertoires contained $(N + m)$ note types. We assigned each new note type a duration that fell between the duration of the note type it was created from and the next longest note type in the repertoire.

We simulated two sets of 2 x $10^4$ populations with different parameter values. In each set, we set $L$ = 4, $g$ = 3, $h$ = 2, $f$ = 0.8, and $m$ = 10. Thus, each population included 60 birds and misclassification errors occurred in 10 note types. In the first set of simulations, we set $N$ = 60 and $n$ = 16. Thus, most birds used only a small subset of note types from the population repertoire. In the second set of simulations, we set $N$ = 24 and $n$ = 48. Thus, each bird used a larger proportion of the note types available to the population.

Table A shows the false positive rates and the powers in our simulations with correct note type classification and with each type of classification error. False positive rates highlighted in red are significantly different from 0.05, and powers highlighted in red are significantly different from those with correct note classifications. When note types were

| parameter values | correct classification | erroneous mergers | erroneous splitting within birds | erroneous splitting among birds |
|---|---|---|---|---|
| $N$ = 60, $n$ = 16 | 0.0504<br>0.6706 | **0.0453**<br>**0.7053** | **0.0569**<br>**0.6906** | **0.0552**<br>**0.6881** |
| $N$ = 24, $n$ = 48 | 0.0493<br>1.0000 | **0.0426**<br>**0.9968** | **0.717**<br>**0.9967** | **0.0694**<br>1.0000 |

Table A. False positive rates (top) and powers (bottom) in 2 x $10^4$ simulations with correct note type classification and with each type of classification error when note type frequencies of use follow a Zipf-Mandelbrot law. False positive rates in red are significantly different from 0.05, and powers in red are significantly different from those with correct note classifications.

erroneously merged, p-values were conservative, and when note types were erroneously split p-values were anticonservative. In some cases, classification errors also affected the power of the test. When note types were erroneously split, the power of the test sometimes appears to increase. This is not surprising, because in this case the test is anticonservative, and thus more likely to indicate ZLA even when there is no relationship between note type duration and frequency of use in the population. It is more surprising that erroneous mergers appear to increase the power in one set of simulations even though the test is conservative. We suspect this is because in this set of simulations each bird used only a small subset of note types, and rare long note types that appeared in the songs of a lineage founder could become disproportionately common in that line. As a result, there was stochasticity in the frequencies with which note types were used in the population. Merging note types may help to reduce this stochasticity. Furthermore, because most birds used only small subsets of the notes in the population repertoire, note types were rarely merged within birds. When the test for ZLA was well-powered, as in the second set of simulations, tests with correct classification outperformed tests with erroneous mergers.

Table B shows the false positive rates and the powers in simulations with correct note type classifications and with each type of classification error when the relative frequencies of use of the note types declined exponentially with the rank order frequencies of use. In these simulations, the $r^{\text{th}}$ most frequently used note type has a relative frequency of use

$$f(r) \approx \frac{k}{exp(z\,(r-1)/(N-1))}$$

where $k = 1/\sum_i exp(-z\,(i-1)/(N-1))$ is a normalizing constant that ensures the relative frequencies of use sum to 1. The parameter $z$ controls the rate at which the relative frequencies of use decline with rank order frequency of use. We set $z = 6$ in the analysis we report here, but obtained similar results for other values of $z$. The results of this analysis are qualitatively similar to those we obtained when note type frequencies of use followed a Zipf-Mandelbrot law (Table A).

| parameter values | correct classification | erroneous mergers | erroneous splitting within birds | erroneous splitting among birds |
|---|---|---|---|---|
| *N* = 60, *n* = 24 | 0.0518<br>0.5203 | **0.0463**<br>**0.5587** | **0.0577**<br>**0.4766** | **0.0575**<br>**0.5400** |
| *N* = 24, *n* = 48 | 0.0512<br>0.9999 | **0.0430**<br>**0.9907** | **0.734**<br>**0.9886** | **0.0695**<br>1.0000 |

Table B. False positive rates (top) and powers (bottom) in 2 x $10^4$ simulations with correct note type classification and with each type of classification error when note type frequencies of use declined exponentially with rank order frequencies of use. False positive rates in red are significantly different from 0.05, and powers in red are significantly different from those with correct note classifications.
