# Supplementary material for: Does Zipf’s law of abbreviation shape birdsong?
Source: PLoS Comput Biol. 2025 Aug 13;21(8):e1013228. doi: 10.1371/journal.pcbi.1013228 (PMC12349147; doi:10.1371/journal.pcbi.1013228)
Supplement: S3 Appendix — (PDF) [file pcbi.1013228.s004.pdf]

S3 Appendix

Songs archived on Bird-DB are annotated at the phrase level. Most phrase types are monosyllabic – each consists of a single sound unit (ie, a note) uninterrupted by periods of silence (eg, phrases a and b in Figure A). However, some phrase types are polysyllabic (eg, phrases c and d in Figure A). These phrases consist of two or more notes separated by short periods of silence. Because these note sequences usually or always appear together in the same order, they were treated as single units in the annotations on Bird-DB [58].

In the body of this paper, we studied ZLA at the phrase level following the annotations on Bird-DB. Alternatively, one might study ZLA at the level of notes. We revisited the song annotations for five populations archived on Bird-DB for which exemplars of each phrase type were available. We examined the exemplar for each phrase type, and if sounds within the phrase were separated by clear periods of silence lasting at least 10 pixels, we divided the phrase type into notes. We classified the notes within the phrase as

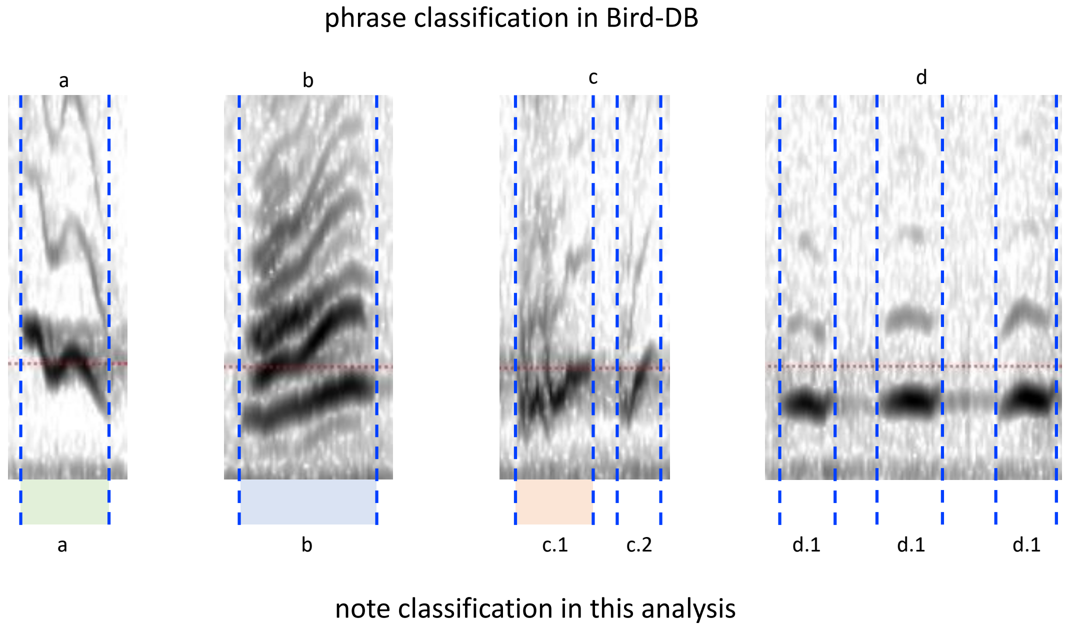

Figure A. Exemplar phrases from Bird-DB reclassified as notes. Phrases a and b are monosyllabic – each corresponds to a single note type. Phrases c and d are polysyllabic. Phrase c is reclassified as two distinct note types. Phrase d is reclassified as three instances of a single note type. Images are phrases produced by California thrashers and archived on Bird-DB [52].

belonging to either the same type (eg, phrase d in Figure A) or different types (eg, phrase c in Figure A). We found no cases where notes created by dividing phrases corresponded clearly to existing monosyllabic phrase types. Therefore, we treated each note type created by dividing phrases as a new note type. We had only one exemplar per phrase type, so we assumed that every phrase of that type could be divided into the same notes, and that the duration of the new notes was proportional to their duration in the exemplar.

We repeated the analyses reported in the body of the paper, and we report the results in Table A and Figure B. The results are qualitatively similar to those reported in the body of the paper. Evidence for ZLA in individual populations is weak, but most populations show trends consistent with ZLA. Concordances are weaker than those observed in written human language.

Because we had only one exemplar per phrase type, our re-classification of notes based on exemplars is likely to be less accurate than the classifications at the phrase level provided in Bird-DB. Thus, we do not encourage other researchers to use the re-classification we conducted here. Nonetheless, the results show that our qualitative results hold under at least some plausible alternative annotations.

species	records (birds) studied	total phrase types	phrases per record	phrase types per record	Shannon diversity	concordance (population)	mean concordance (individual)	maximum significant concordance
California thrasher	89	809	160.1	15.4	2.28	**-0.071 (p = 0.002)**	-0.008 (p = 0.359)	-0.038
	7	187	423.0	59.1	3.66	-0.073 (p = 0.075)	-0.073 p = 0.063)	-0.081
black-headed grosbeak	83	481	165.7	29.1	2.98	0.002 (p = 0.527)	**-0.051 (p = 0.042)**	-0.049
Cassin's vireo	13	71	93.5	28.0	3.02	0.097 (p = 0.878)	0.015 (p = 0.577)	-0.137
	41	119	101.1	28.3	2.92	-0.007 (p = 0.458)	-0.056 (p = 0.089)	-0.068

Table A. Summary statistics and concordances in the songs of 5 populations of 3 bird species archived on Bird-DB. Polysyllabic phrases in the original data have been separated into their component notes. P-values less than 0.05 indicate patterns consistent with ZLA (bold red).

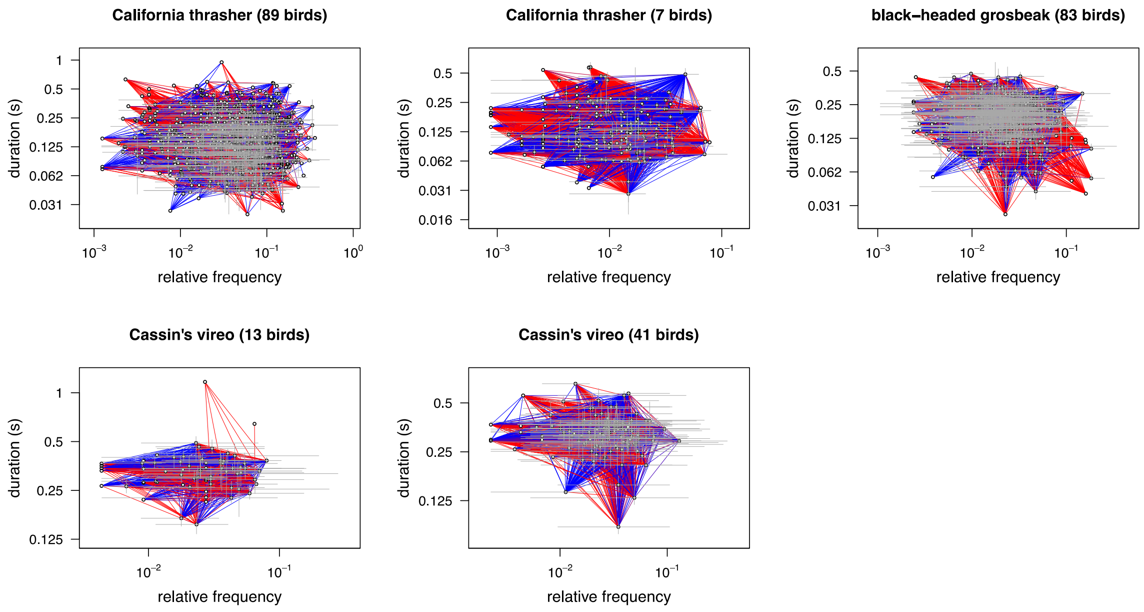

Figure B. Concordances between reclassified note type durations and frequencies of use in 5 bird populations. Each open circle represents the mean frequency of use and the mean duration of one note type used by the population. Horizontal (vertical) grey lines show the range of relative frequencies of use (durations) among birds that used each note type. Note types that appear together in the repertoire of at least one bird are connected by colored lines. Heavier lines indicate that the pair of notes was used by more birds. Blue (red) lines indicate that the concordance between frequency of use and duration was positive (negative) for that note pair. Intermediate colors indicate that the concordance was positive in some birds and negative in others. For example, this can occur when some birds use the note type more frequently than other birds. ZLA is present when the concordance between frequency of use and duration is negative. Thus, we should expect to see more red lines in figures for populations that adhere to ZLA.
