# Supplementary material for: Does Zipf’s law of abbreviation shape birdsong?
Source: PLoS Comput Biol. 2025 Aug 13;21(8):e1013228. doi: 10.1371/journal.pcbi.1013228 (PMC12349147; doi:10.1371/journal.pcbi.1013228)
Supplement: Attachment — Submitted filename: Author Contributions for PLoS Comp Bio.docx [file pcbi.1013228.s005.docx]

Author contributions are not included in the manuscript we submitted to PLoS Computational Biology directly from bioRXiv. We wish to add the following author contributions to the submitted manuscript:

**Author Contributions**

RTG and RNL conceptualised the study. RTG and LM collected and processed the data, and RTG and CDD analysed the data. RTG wrote the first draft of the manuscript, and all authors revised the manuscript.
