# Supplementary material for: Does Zipf’s law of abbreviation shape birdsong?
Source: PLoS Comput Biol. 2025 Aug 13;21(8):e1013228. doi: 10.1371/journal.pcbi.1013228 (PMC12349147; doi:10.1371/journal.pcbi.1013228)
Supplement: Attachment — Submitted filename: Draft notes AM.docx [file pcbi.1013228.s006.docx]

#### Dr Andrea E Martin and Prof Natalia Komarova

#### Academic and Section Editors

*PLoS Computational Biology*

27 May 2024

Dear Dr Martin and Prof Komarova,

My colleagues and I are grateful to you and three anonymous reviewers for taking the time to read and comment on our work. We found the reviewers’ comments to be insightful and constructive. We have revised our manuscript to reflect their helpful advice, and we believe the manuscript is clearer and better as a result. With this letter, we are submitting the revised manuscript for your consideration.

Our resubmission includes two copies of the manuscript – a copy with changes tracked so you and the reviewers can easily see where we have made revisions, and a clean copy that may be easier to read. We have appended to this letter our responses to the reviewers’ comments, with the reviewers’ comments in black and our responses in green. All line numbers in our responses refer to lines in the document with tracked changes showing.

We thank you again for your consideration.

Sincerely,

R. Tucker Gilman

Senior Lecturer in Environmental Research

Department of Earth and Environmental Science

University of Manchester

tucker.gilman@manchester.ac.uk

Reviewer #1: This paper asks whether Zipf’s law of abbreviation (ZLA), which is ubiquitous across languages, is present in birdsong as well. ZLA is based on the information theoretic concept that to most optimally convey information, more frequently used vocalizations will be shorter. Because ZLA describes optimal behavior in conveying information, and both the information content and units of information (e.g. syllables vs phrases) in birdsong are not well understood, this is a challenging problem, and the literature varies in whether ZLA is observed or not. Additionally birds generally produce unique vocal repertoires, thus individual identity needs to be taken into account when computing ZLA. To solve this problem, the authors implement a previously reported method for assessing ZLA in birdsong as an R package and test it on birdsong in the bird-db repository. They generally do not find any evidence for ZLA in the datasets, making the main analyses a null result. In the context of the current literature on ZLA in songbirds, this paper advances the field by implementing a common R library based on prior methods, but the actual analyses do not provide much information about whether ZLA exists in birds or not. The general conclusion is that this is hard to assess, but the authors never directly address why it is hard to do in birdsong - this seems like an approachable task given the methods they developed for the paper - for example looking at how many birds, how many notes/phrases, and how many unique notes/phrases are needed in a synthetic repertoire to observe LDA and comparing their empirical birdsong datasets to those measurements.

Researchers study ZLA by looking for negative concordances between note type durations and their frequencies of use. Doing this in birdsong is difficult for several reasons. The most important is that note type repertoires within individuals and populations are often small, at least relative to the numbers of words in human languages. In general, the ability to differentiate an observed concordance from those that might arise by chance under the null hypothesis depends on the size of the note type repertoire. Or, put another way, the sample size for these analyses is closely related to the number of note types. For populations with extremely small repertoires, like Favaro and colleagues’ (2020) African penguins, even a perfect concordance is only weak evidence for ZLA. Biologically plausible strengths of ZLA may be difficult to detect in many or even most bird populations, and if note type repertoires are small then no amount of additional study will solve the problem for individual species. For this reason, it may be that the only way to assess ZLA in birdsong generally is to study many populations and ask if weak trends in the direction of ZLA are more common than we would expect by chance. We explain this in lines 120-130 of the introduction. The R package we present with this study will make this work possible.

In this context, the idea the reviewer raises above is interesting and important, and the other reviewers made similar suggestions. To synthesise the comment/question across the reviewers, “If ZLA exists at a plausible strength in birdsong, should we expect to detect it in the populations we studied, or in other populations we might study?” Reviewer 1 suggests that we could answer this by studying simulated data, and asking how many birds and note types we might need to detect ZLA. We think this would be very difficult. As the reviewer suggests, the answer would depend on individual and population repertoire sizes and on how similar the repertoires are among birds, but it would also depend on what the “plausible strength” of ZLA is and on repertoire properties like the relative frequencies of the note types and which specific note types are more frequent than which others. For example, we might get the same strength of concordance because a few very short note types are very common, or because many slightly short note types are a little more common than average. Studying heuristic datasets can be convincing if the goal is to derive qualitative inferences about false positive rates and relative analysis power under different kinds of note classification errors that might arise. It would be less convincing if the goal were to make quantitative inferences about analysis power in real populations that exist but about which we know very little.

Fortunately, we don’t have to simulate data to address the reviewers’ question, because we can answer it using the observed data. When we compute null distributions for concordances in our analysis, we necessarily obtain significance thresholds for the analysis – that is, we obtain the least negative concordance that we would interpret as evidence for ZLA. So, while we cannot report how many notes or birds a particular population would need for us to find evidence of ZLA, we can report how strong the concordance would have to be for us to find statistically significant evidence of ZLA in the populations we have. We have done this in our revision, and added this information to tables 1, S2.1, and S4.1. We have also updated the ZLAvian package to report this information to users.

Overall, this new information shows that concordances would have to be stronger than those we observed for us to report statistically significant evidence for ZLA in the populations we studied. We knew that of course, because we did not find evidence for ZLA in our individual populations. More interestingly, the results suggest that we could detect ZLA in many bird populations with concordances weaker than ‑0.1, which is about half the strength of the negative concordance reported in human languages, and weaker than concordances that have been found in at least some birds (see Youngblood 2024 and western tanagers in this study).

Specific points
I found the way that the authors motivate the expectation for ZLA in birdsong confusing and at times circular. ZLA proposes that common vocalizations will be shorter to maximize information. In birdsong, information contained in a song is not well understood. In this study, non-song vocalizations are removed from analysis, so the data is purely composed of birdsong. The authors motivate this analysis by saying that when a male is singing to a female, the information that is contained is fitness - longer and more complex vocalizations signal fitness, which is well established. But if the information conveyed in a song is tied to how long a vocalization is, then the expectation should be that ZLA does not exist in birdsong. But this is not conveyed.

We agree with the argument the reviewer presents here, and we tried to convey this same idea in the introduction to our original submission. The importance of this argument, in our opinion, is that it highlights why the search for ZLA in birdsong is interesting, and also why the ZLAvian package is important. Some authors have argued that ZLA should arise in birdsong, and some even claim to have found it (eg. Favaro and colleagues 2020, Youngblood 2024). But, there is an equally strong argument that ZLA should not arise in birdsong, and other authors have not found it (eg, Lewis et al 2023, Ferrer-i-Cancho and Hernández-Fernández 2013). Theory cannot resolve this debate, so we will have to turn to the data, and that is the motivation for this study. We have edited the introduction (especially lines 116-118) to make this clearer.

The above point is related to the author's notion of “intent”, which is discussed but not defined. This seems fundamental to a question about the relationship between information-carrying units of birdsong and frequency. For example, if we were to segment speech into phonemes, syllables, or phrases, we presumably would not observe ZLA. The authors start to get at this issue by looking at different levels of organization for birdsong - many of the annotations in their dataset are composed of phrases rather than syllables, so they come up with a secondary way of doing the analyses by segmenting phrases into syllables. It seems the value of this analysis is in sweeping the range of possible ‘informative units’ in birdsong to see whether one conforms to ZLA or not. This second analysis is also mostly null, however, with similarly small effects in positive examples.

The reviewer is correct. We focused on analyses at the level of phrases (the original classifications archived in Bird-DB) because these were assigned by experts on the bird species whose songs they classified, and are thus the “gold standard” for each system. We repeated the analysis with our own re-segmenting of phrases into notes (syllables). We are less confident in our re-segmenting. Mover, in principle, if two note types always appear together then it makes sense to treat them as a single unit for the purpose of compression, and thus for studying ZLA. Nonetheless, we cannot know absolutely which is the more appropriate level for detecting ZLA in any given population. Happily, the results are qualitatively similar (lines 425-428 and supporting information S4). So, our story does not change depending on which level we choose.

I see two major contributions of this work. The first is that the authors are taking a question that has been asked on a number of individual species and looking at a broad sampling of birds to see if a general trend emerges. The second is that the authors are presenting a statistical method and R package for looking for ZLA in symbolically segmented data like birdsong. For the large sampling approach - birddb, while a valuable resource, contains relatively small datasets sampled from the wild - would there be value in adding some more species, e.g. bringing in the data from the other species mentioned in the introduction?

We agree that there is value to be had in applying our analysis to other species! That is why we provide the ZLAvian package, and we are pleased that the reviewer sees this as a major contribution. We are not sure that the populations mentioned in the introduction will necessarily be the best ones to study. In some case (eg, Favaro and colleagues’ (2020) African penguins), the repertoires are very small. In others (eg, Connor’s (1985) ravens), the original data is not available and it would be difficult or impossible to assign repertoires to individuals. However, many labs and researchers have datasets from a range of species that could be analysed using our method. Aggregating and analysing that data is beyond the scope of this study, but we believe that the ZLAvian package will make such analyses easy. We anticipate that this will lead to new evidence for or against ZLA in birds and potentially other animals.

For the R package approach - for this to be a valuable resource the paper would need to additionally include a link to an online repository and online documentation (and might be better suited as a separate paper to something like JOSS).

There is link to the ZLAvian package, with documentation, in line 275. We thought this link was working properly at the time of the initial review, but perhaps it was not. We have tested it again here and can confirm that it is working now. A main objective of this paper is to introduce ZLAvian – in fact, that is why we submitted to *PLoS Computational Biology* rather than to a more specialist journal - so we don’t think another strictly methodological paper is needed. However, as reviewer 3 observed, more formalisation was needed to properly introduce ZLAvian here, and we have added that in Box 1.

A figure plotting the relationship between frequency and duration should be in the main text. For example, appendix 3, but for both notes and phrases.

Following the reviewer’s advice, we have moved the figure from appendix to the body of paper (Figure 1). In the interest of conserving space, we have done this only for the analysis at the level of phrases. As explained in lines 425-428, the phrase-level and note-level analyses offer qualitatively similar results, and because the phrase-level classification was done by experts on the populations being studied, we believe that the phrase-level analysis is more convincing. However, following the reviewer’s advice here and above, we have added similar figure for the note-level analysis to the supporting information (Figure S4.2).

Why is alpha set at 0.1 for significance?

All the reviewers asked this question. We did this because ZLA is consistent with a negative concordance between note type duration and frequency of use, and we wanted to have the conventional 5% false positive rate for detecting ZLA. But, at the population level we sometimes found surprisingly strong *positive* concordances between note type duration and frequency of use, and we wanted to draw attention to those. If we consider both 5% tails of the null distribution to be significant, then our combined false positive rate (that is, the probability of getting either a false positive or a false negative) is 10%, so alpha is 0.1.

In retrospect, we think that this decision was confusing and unnecessary. We report the precise p-values for our tests of ZLA in the populations we studied. Those p-values give readers enough information to decide how much credence to put in our results. So, there is no reason to impose an arbitrary significance threshold. And, given that the p-value for ZLA is less than 0.05 in only one of the populations we studied, the strongest evidence we have for ZLA in birdsong is the preponderance of weakly negative concordances (10 of 11 populations). This pattern does not rely on significance thresholds.

In the revised manuscript, we reserve “significant” for the one-tailed hypothesis test that concordance is more negative than we would expect under the null (ie, consistent with ZLA). However, we still point readers to patterns that are strongly inconsistent with ZLA where they arise in our study (see lines 456-461 and 494-496).

The library/methodology the authors developed should be validated with synthetic data and language data beyond misclassification errors - for example, determining theoretical data requirements for observing ZLA in a dataset of birdsong. On that note - how many unique note / phrase types are needed at minimum to do this analysis? Do all of the species meet these requirements?

Please see our response to this reviewer’s first comment. The number of note types needed to detect a “significant” concordance between note type duration and frequency of use depends on the strength of the concordance. In principle, if the concordance were perfect, 5 note types would be enough (provided that at least some birds used all 5 types). All the populations we studied all had at least 9 phrase types. In practice, concordances are not perfect and in most populations each bird uses only a subset of the note types in the population repertoire, so the number of note types needed to detect significant patterns will vary among populations. In this revision, we report the minimum strength of concordance that would be detectable (at alpha = 0.05) in each of the populations we studied given the note types it used (Tables 1, S2.1, and S4.1). We have updated the ZLAvian package to provide this information when future users analyse their own data. We believe this is the most robust way to address the issue the reviewer raises with this comment.

Are we limited by the amount of data in these datasets? How did you decide when not to include an animal (i.e. when there is too little data for an animal). This is a recurring point in the text but is never addressed in a satisfying way.

If a bird uses more than one phrase type, then we can compute the concordance between the phrase type durations and the frequencies of use in its repertoire. So, we can obtain information from that animal. Unless an animal uses only one note type, there is no reason to exclude it from the analysis (lines 433-437). However, our analysis weights birds more heavily if they use more note types, because concordances based on more note types are more accurate representations of the behaviour of the animal. We explain this in lines 317-323 and in lines 819-822 in Box 1. See also our response to a similar comment from reviewer 3.

The lack of figures and sectioning generally made it hard to follow the paper. It would aid the reader to see clear sections with supporting figures. For example, a structure where data is presented, models are defined, synthetic analyses are presented, and empirical data are analyzed. Currently the lack of supporting figures requires the reader to ‘take your word for it’ on a lot of the analysis.

We have restructured the introduction and methods sections with clearer sections in this revision, added more signposting for readers (eg, lines 298-301), and moved the key supporting figures from the supporting information to the body of the paper. We believe this makes the paper easier to follow and thank the reviewer for the advice.

Reviewer #2: This paper introduces a method to test whether birdsong confirms to Zipf’s Law of Abbreviation offering an accompanying R package. The paper is well written, and the methods and results are described clearly. It tackles a question that has been addressed by previous research although with somewhat conflicting results. Although the question is not novel and the research is not very original, there is definitely value in its approach, especially because it offers a way for other researchers to answer the question with their own data sets.

We are pleased that the reviewer sees value in our work, and reluctant to quibble over minor points. However, while the question of whether ZLA appears in bird vocalisations has been asked before, we think our work makes important and novel contributions to *how* that should be done. In particular, we show that concordances between phrase type duration and frequency of use can be different depending on whether we study them at the population level or at the individual level. This is important because most previous work has studied trends at the population level, but Zipf’s principle of least effort predicts trends at the individual level. To our knowledge, this difference has not been appreciated in studies of animal communication. Furthermore, the question of how note misclassifications – and especially misclassifications that are systematically biased relative to note durations - might affect inferences about ZLA has not, to our knowledge, been discussed. So, we think our work does more than contribute new evidence to an open question – we think it will change the way the question is asked in the future. In that respect, we believe it is novel. We hope that the streamlined introduction in our revised submission will help to highlight these ideas.

Not being a computational biologist myself, I am not qualified to comment on the appropriateness and details of the model. It seems straightforward enough to me, and as far as I can tell, there are no major issues with it.

I am going to comment on other aspects of the paper.

I have a basic issue with the study, which is that motivation for doing it in the first place is not well established. It struck me that the authors not only spent a long time discussing why it is difficult (even impossible in many cases) to classify and measure components in birdsong to test whether they conform to patterns predicted by ZLA, but they also detailed, pretty convincingly, the case against ZLA, why it probably wouldn’t make sense for it to evolve in birdsong. This is because longer and more complex songs have been shown to be appealing to females, and males who sing them often and loud will probably produce more offspring and will have more juveniles copying their songs (as male offspring tend to imitate their father’s or close relatives’ songs more than strangers’ songs). This would result in strong evolutionary pressures against short or simple songs and song syllables. With this in mind, the authors did not make a convincing case for doing the analysis, they say “Nonetheless, we believe such data is worth analysing” which does not seem like a good enough reason. I do agree that it’s interesting to look for commonalities between birdsong and human language, but there must be a strong conceptual motivation for it which has not been established in this paper.

There is indeed theoretical reason to question whether ZLA should arise in birdsong, and we are happy that the reviewer found our argument for that convincing. However, the theoretical argument is counterbalanced by empirical evidence consistent with ZLA, from Favaro and colleagues (2020) and more recently from Youngblood (2024) in a population with a much larger repertoire. Furthermore, while the empirical results we present here are not convincing evidence of ZLA in any given population when studied alone, we find a trend in the direction of ZLA across many different populations. In that respect, our argument that ZLA might not rise in birdsong is something of a straw man – one might expect it to be true, but it is not clear that it is. For us, this makes the question of whether ZLA exists in birdsong not just open but also exciting. If it were not, there would be little value in introducing an R package to study it! In our revision, we have emphasised that whether birdsong adheres to ZLA is an open and exciting question in lines 106-118 (especially in 116-118).

Following on from the previous, I think it would have made more sense to exclude song syllables and focus only on calls which are used by both males and females and are much more dynamic and flexible in their use than songs. Moreover, songs are believed not to carry any sort of sophisticated meaning (besides “mate with me”) while calls probably convey more subtle social meaning, making them possibly more similar to words in human language. I don’t know if it’s possible to separate songs from social calls in the sample used in this study, but as it is, it seems to me that there isn’t a strong enough case for doing the analysis on the chosen sample.

We agree with the reviewer than bird calls may be a valuable system for studying ZLA in animal communication, but doing this may require data that does not yet exist. We now discuss this in lines 604-618. The tools we provide here will facilitate that work when data become available. In the meantime, the needed to study ZLA in bird calls does not mean we *shouldn’t* study ZLA in birdsong. As the reviewer implies, they are different systems, and as we explain our previous response, the question of whether birdsong adheres to ZLA is interesting and open.

I also wondered why the authors used songs that were annotated by hand when, as they acknowledged, sophisticated cluster analysis methods already exist for the classification of notes. Of course, these methods are not fool-proof either and subjective decisions must still be made about what features constitute a note or phrase, but the annotations used in this study seemed to contain variability which forced the authors to have to split up songs within a species into separate populations.

Computational algorithms for classifying notes in birdsong exist and are rapidly improving, but at the current time classification by expert inspection is still the gold standard. Indeed, supervised clustering algorithms (eg, Kaewtip et al 2016, Cohen et al 2022) still rely on human-classified notes for training data. Unsupervised algorithms also exist (eg, Lachlan et al 2018). However, an important concern for unsupervised classification algorithms is that, currently, we have only a poor understanding of how the classifications they achieve relate to the acoustic characteristics of the notes being classified. For example, we do not know exactly how particular clustering algorithms respond to differences in note durations when classifying notes. This may be a particular problem for studying ZLA. For example, imagine a population repertoire in which the variability in duration among individuals when they produce a particular note type scales with the expected duration of that note type in the population, as seems to be true in many species (see Lewis et al 2021 and Supporting Information S1 in this study). If a classification algorithm separates notes into types based in part on the untransformed difference in their durations, then that algorithm will systematically over-split longer note types. Then, there will be patterns consistent with ZLA in the note types as classified by the algorithm even if there were no such patterns in the “true” note types the birds intended to produce. Indeed, there is at least some reason to think that this may happen. When Youngblood (2024) computationally classified notes using dynamic time warping and hierarchical clustering (following Lachlan et al 2018) and studied those notes using ZLAvian, his inferences about ZLA changed qualitatively depending on how aggressively he allowed the classification algorithm to split groups. In the future, the computational classification of notes may match and eventually exceed the accuracy achieved by human experts, and we hope that researchers who produce these classifications will use ZLAvian to study them. However, for now, the most robust classification is still by human experts. A broad study comparing inferences about language laws obtained using human and computationally classified note types might be a valuable contribution to the field, but we believe that is another study.

In passing, we note that while reclassifying phrases might allow us to merge populations with songs archived on Bird-DB, it is not clear that we *should* do that. Different bird populations can have different dialects, and strengths of concordance might differ among them in interesting ways. These differences would be lost if we studied ZLA in species instead of in populations.

Some of the species contributed only 2, 3 or 4 records, this seems too little for the analysis attempted by the authors. There was huge variation in the availability of data between the different species and populations, and this problem was not sufficiently addressed in the ms.

This is an interesting point. If we detect a trend consistent with ZLA in a very small sample from a population, then we cannot say with confidence whether that trend is common in the population or whether the trend in rare and we just happened to sample a few rare birds that exhibit that trend. If we detect the same trend in small samples from many populations, as we did here, then we still may not be able to say with confidence whether the trend is common or rare in any given population, but across all populations the trend is likely to be common, because we would be unlikely to sample just the rare birds that adhere to the trend from many populations independently. This argument is analogous to the argument that weak trends in the direction of ZLA found in many different bird species and populations can eventually add up to compelling evidence for ZLA in birdsong as a whole. That is, while p-values tell us how confident we are that a trend we observe is stronger than we would expect by chance, large samples offer confidence that the observed trends are likely to be general. We agree with the reviewer that this was not adequately addressed in the original submission, and we now discuss this in lines 524-532.

It is not clear why the authors used an alpha level of 0.1, and the interpretation of p values is very unusual. What was the null hypotheses for the tests? Not enough information is given on the statistical tests.

We agree that this was confusing, and we have changed the way we approach this throughout our revision. Please see our expanded response to the similar comment from reviewer 1, and lines 456-461 of the revised manuscript.

I was also a bit confused by the treatment of alarm calls. The authors excluded repeating notes from their analysis because, as they say, these may represent alarm calls that probably adhere to different rules than songs or calls. However, in the discussion, they make the point that alarm calls may make appealing targets for ZLA analysis. This contradicts the earlier decisions, and I wasn’t sure how to interpret it.

We agree with the reviewer that the discussion of alarm calls in our initial submission was confusing.

Some birds with annotations in Bird-DB produced only one phrase type. We cannot compute within-bird concordances for these individuals, because concordance requires at least two phrases. So, we can exclude these birds from the analysis without losing information. Additionally, recordings with just a single phrase type are likely to be (but may not be) alarm calls. Calls may follow different structural and compositional rules than songs, and should not be analysed together.

Despite the above, studying ZLA in bird calls may be useful. Calls may have lexical meaning, so we might expect ZLA in bird calls to arise in the same way it does in human languages – something we may not expect in song. At this time, neither Bird-DB nor any other existing dataset that we know of contains the data needed to study ZLA in bird calls, but we hope this data can be collected in future work. We now explain this in lines 604-618.

I appreciate that the authors were open and honest with the limitations of the study and the general approach, but the motivation, the sample and the analysis should have been better explained in the ms. All of these areas feel minimally addressed in the current draft.

We thank the reviewer for the advice. We have addressed the reviewer’s specific comments above, and we think the explanations in the paper are much improved as a result.

Reviewer #3: This is very timely research as linguistic laws remain underexplored in birds (leaving aside penguins and ancient work by Hailman that needs to be revised as the authors correctly argue). In addition, bird song is crucial step as it deviates from previous research in the sense that it points to phonological syntax rather than to lexical syntax in Marler's sense. To the best of my, knowledge, this is the widest exploration of Zipf's law of abbreviation in bird song that has ever been performed!
The article report weak support for the law of abbreviation (for most species, the sign of the correlation is negative as predicted by the principle of compression; however, the correlation is non-significant in most case). The article has various strengths: the wide range of species considered and the concerns about the impact of categorization on the emergence of the laws. However, I think that the clarity, methods and accuracy of arguments can be improved. I would love to see a substantially rewritten version. This article has the potential to become a milestone article for research in bird song and it is likely to boost research on linguistic laws within the bird song community.

Thank you! We are pleased that the reviewer believes our work is timely and can become a milestone and advance future work in the field.

p. 7-9 introduce "A method for assessing Zipf’s law of abbreviation in birdsong" but not in the methods section but in the introduction. Thus authors try to present critical methods following the literary style of introductions. These pages are hard to understand because the authors have minimized the use of mathematical notation and replaced complex formulae by verbal explanations. These explanations are ambiguous and critical detail is missing. This method must be clearly explained in the methods section (with formulae and mathematical notation to guide explanations with precision). In the introduction, the authors may retain the essential information about the method. With the current vagueness of these pages, the results are difficult to interpret in depth.

That methodology is presented in a paper by Lewis et al that the authors cite. However, a reader of the present article should not be forced to read Lewis et al's article (ref 35) first. Lewis et al's article should be a back-up but not a must read. Having said that, I am totally sympathetic with the authors because I understand their challenge (I have faced that challenge in the past). I am sure they can fix it.

We agree, and correcting this issue has been an important focus of this revision. The challenge is that this section of the paper must achieve two things: it must explain *what* we did, and it must explain *why* we did it. Both of those explanations must be unambiguous. Understanding mathematical and statistical analyses precisely is often easiest if the explanations are formalised into clear equations. But as readers, we (the authors) often find mathematically intensive explanations difficult to follow if they are frequently interrupted by complicated rationales. So, in our original submission, we attempted to present our method in a narrative style in the introduction without equations. This attempt was not fully successful.

In the revision, we have addressed this in three ways. First, we have moved the description of the analysis to the methods section as the reviewer recommended. Second, we have added or rewritten large sections of that description to clarify elements of the analysis that we thought were unclear or to address questions raised by the reviewers (lines 302-399). Finally and most importantly, we have added a text box with a formal and mathematically precise explanation of the method (lines 813-844, but this will appear with the methods section in the type-set paper). Thus, readers can use our narrative to understand broadly how the analysis works and visit the text box to confirm their understanding of the maths, or alternatively can use the text box to understand precisely what the method does and then return to the main text for a clearer explanation of why. We believe this is a major improvement to the paper, and we thank the reviewer for the advice.

Zipf's law of abbreviation is an inductive pattern and thus it does not predict anything in a strict sense. It is important to distinguish between principles and their manifestations in the abstract and other parts of the article. The compression principle predicts Zipf's law of abbreviation. Zipf's law of abbreviation is one of the manifestations of the principle.

Yes, we agree, and thank the reviewer for the clear explanation. We have corrected this in each place it occurred in the original submission. (There were many, so we have not listed all the line numbers.)

p. 2 "Zipf’s law of abbreviation (ZLA) states that, in human languages, words that are used more frequently tend to be shorter than words that are used less frequently." a reference to Zipf's (1949) classic book is required.

We agree and have added the reference (lines 70, 73, 514, 555).

p.5. "Birds that use longer note types sing fewer notes in each song. In such species, if birds that sing shorter note types are at least as common as birds that sing longer note types, then we might see patterns consistent with ZLA at the population level even if no individual bird uses short note types more frequently than it uses long ones. However, such a pattern would not provide evidence for the principle of least effort proposed to underlie ZLA."

"Birds that use longer note types sing fewer notes in each song" This seems an example of Menzerath's law (or Arens' law), which can be interpreted as manifestations of the principle of least effort (see Ref 30 and Gustison et al 2016 for the theoretical arguments). Lewis and collaborators have already investigated that law.

This is a very interesting observation. In human languages, Aren’s law can be measured inter-textually (by computing the mean word length and sentence length in each text and then measuring the pattern among texts) or intra-textually (by computing the concordance between word length and sentence length among individual sentences within texts). The patterns that arise inter-textually need not be the same as those that arise intra-textually, and they may arise due to different mechanisms. Some authors have argued that in human language, Menzerath’s law should be measured only at the intra-textual level (Grzybek and Stadlober 2007). It is not clear what units of birdsong (if any) equate to texts, but whatever those are, the repertoires of different birds would almost certainly represent different “texts.” So, our observation that birds that use longer note types sing fewer notes in each song is analogous to an intertextual analysis, and some readers might reasonably debate whether this is really Menzerath’s law. We think it would be better not to wade into that debate in this paper. However, there may be exciting work to be done on this question in the context of animal communication!

That said, we are grateful to the reviewer for this comment, both for the idea and because it led us to Grzybek and Stadlober’s (2007) interesting paper, which we now reference in lines 558-559.

[METHODS]
p. 7 "We compute the mean logged duration of each note type as produced by each bird in the sample, and we count the number of times that each bird produced each note type." The median is considered to give a more robust summary of the durations. See section 4.1 of Petrini et al 2023 for a justification based both on research on humans and other species. The authors should justify the use of the mean and check if their conclusions would change and be more in line with the prediction of compression if means where replaced by medians.

This is an excellent idea. We now explain why we chose to work with means rather than medians in lines 391-396, and we confirm that the results are qualitatively (and almost quantitatively!) similar if we study medians instead of means in part 2 of the supporting information. Moreover, we have updated ZLAvian so that users now have the option of working with medians instead of means.

The motivation of using logged duration instead of raw duration is unclear (logged duration). The Kendall tau correlation score will give the same value regardless of whether durations have been logged or not.

Our observed Kendall’s tau will be the same if we work with raw or logged durations, as the reviewer correctly observes. However, the null distributions for tau will be different. We now explain this in lines 371-391. (The explanation is long and somewhat complicated, so we have not reproduced it here.) As we now explain in the text, logging is appropriate when duration is used as the measure of effort invested in notes or phrases, but may not be appropriate for some other measures of effort that researchers might want to work with (eg, see Youngblood 2024). Therefore, we have updated ZLAvian so that users now have the option of working with either raw or logged values (lines 396-399). We thank the reviewer for the advice.

p. 7. "We compute \bar{\tau}, the population mean value of \tau with each bird weighted by the inverse variance of its \tau." Precise equation needed. I do not understand the meaning of inverse variance of \tau.

The tau we observed for each individual is a random variable, and its variance is related to size of the repertoire for which it is computed. To see this intuitively, consider that there is some expected tau for birds in the population. Regardless of that expectation, a bird that uses two phrase types can have only one of two taus: -1 or 1. If a bird uses 100 phrase types, its tau can (and probably will) more closely represent the expectation for the population. The most accurate estimation of the expectation for the population can be obtained from a weighted average where each observation is weighted by its inverse variance. Of course, the reviewer is correct that an equation and proper explanation are required. We now provide those in lines 318-321 and in Box 1.

p. 8. "(\bar{\tau} + 1)/2 is the probability that the longer note type would appear more frequently." I am familiar with the mathematics of rank correlation but I cannot follow the argument. Kendall tau correlation is the probability that a pair of points in the sample are concordant. The authors are stating that half the probability that two pairs are concordant + 0.5 is the probability that the longer note appears more frequently (for simplicity I have assumed Kendall tau a; Statistical packages such as R using Kendall tau b, which is a renormalized proportion).

I think that even a mathematically oriented reader needs help. Mathematical statements should be justified properly. Detailed arguments can be put in an appendix. Be careful with solutions that are just point to another paper where the lack of a proper mathematical argument remains.

Our understanding of Kendall’s tau is slightly different from the reviewer’s description. In the form we are familiar with, Kendall’s tau (both a and b: the difference is that tau b includes a correction for dealing with “ties”) is a concordance rather than a probability. It ranges from -1 to 1, whereas probabilities are bounded at 0 and 1. However, concordance is a linear function of the probability that the measures of randomly selected points are concordant, and the formula in our paper converts the concordance to that probability. Nonetheless, we agree with the reviewer that readers need enough information about formulae to understand why they are used, and we have added than in lines 324-328.

p. 8. Second paragraph. I do not understand the null model. \tau should have an expected value of zero. Tau has an expected value of zero when one of the columns of the matrix is shuffled at random.

Indeed, tau has an expected value of zero, and simply shuffling one column of the matrix for each bird would produce a null distribution for tau. But, it would not be the correct null distribution for the hypothesis we want to test. Our hypothesis is that note duration and frequency of use are negatively concordant within birds, and we want to test this while controlling for the fact that birds learn their songs from others in their populations (ie, the songs of individual birds are not independent). So, when we shuffle durations among note types, we need to apply the *same* reshuffling in every bird. That is, if the duration from note type A is paired with the frequency of use of note type B in one bird, then the same pairing must be assigned in every bird. Thus, the frequencies of note types within birds and the shared note types among birds are maintained even as the durations of the note types are shuffled.

Furthermore, our null hypothesis must account for the fact that birds produce note types differently, sometimes to the extent that the rank orders of note durations differ among birds. So, it is not enough to shuffle the population means for note types, we must also reassign the interindividual variability to the note types after the means are shuffled. The interindividual variability must be reassigned in a structured way because, as with the mean durations, interindividual variability may be learned and thus non-independent among birds – eg, fathers and sons may deviate from the population mean in the same ways across many note types. Nonetheless, the reassignment of interindividual variability must also be independent of the frequency of note type use. Adding or subtracting the observed deviance matrix from the huffled note durations achieves these goals.

Obtaining the null distribution is more difficult than the reviewer’s comment suggests, but the importance of the comment is that the reviewer made it, which shows that we were not successful in explaining our approach in the original submission. In the revision, we have rewritten large parts of the methods describing how we obtain the null (lines 330-367) and added the formalisation in Box 1. We think this makes the rationale behind the approach clearer. We thank the reviewer for helping us improve the manuscript.

p. 9. I do not understand. Verbal explanations are imprecise.
One should not have to read the article by Lewis (ref. 35) to be able to follow the current article.

We agree, and apologise for sending the reviewer on a scavenger hunt for our methods. We now provide a mathematical formalisation in Box 1.

p. 10 and other places. The authors confront bird song against research in humans in ref [15] on written language. It would be useful to cite research on the law of abbreviation in spoken language, e.g., Petrini et al (2023) and across linguistic units, not only words (Hernandez-Fernandez et al 2019, Torre et al 2019).

We now refer readers to this work in the introduction (lines 75-76) and elsewhere (lines 392, 570). We thank the reviewer for the advice.

[METHODS]
p. 13 the comparison of bird song (duration of vocalization) versus human language (word length) is weak in the sense the modality is different (vocal versus written) and the units of measurement (duration/time versus length in characters) is very different. There are two ways of addressing this: (a) using oral language and durations (see for instance Petrini et al 2023 or articles by Torre ) or (b) acknowledging the limitations of the control and nuancing the conclusions across the article. As reviewer, I am fine with just an accurate application of (b). Carrying out (a) controlling for speaker variation (the identity of the speaker is unknown in certain dataset; certainly not in Petrini et al 2013) and covering a sufficiently wide range of languages can be exceedingly complex.

This is closely related to a comment the reviewer made below about text on page 17. We agree in both cases. In this revision we have been more cautious about interpreting our comparison of ZLA in birdsong and human language. (This required revisions throughout the paper, from the abstract to the discussion, so we have not provided a full list of edits in response to this comment.) We agree with the reviewer that a formal comparison of ZLA in birdsong and human speech is beyond the scope of this paper.

In addition, I have not seen that the authors have controlled by number of tokens (the correlate of raw number of phrases in their analysis). The questions is if Zipf's law in humans becomes as weak as in birds when the same sample size (number of tokens) is the same as in birds. It is customary in quantitative linguistics to check if the findings still hold when using samples of same size for a fairer comparison (a subtle point is that it can be argued that using only samples matched by number of tokens is not enough due to the scaling properties of symbolic sequences, e.g. a natural language text of 100 words may not have the same statistical properties of a text of 10000 words).

In this comment the reviewer questions how differences in ZLA between birdsong and human language were (or should be) assessed in our study. In a comment below (p. 17), the reviewer raises concerns about how we interpreted those differences. The comments are related so we address them together here.

We believe that the most appropriate way to compare ZLA across systems (eg, among bird species, or between birdsong and human language) is not by comparing the p-values associated with the hypothesis tests for ZLA in the systems, but rather by comparing the test statistics (ie, the $\bar{\tau}$s) in the systems. As the reviewer rightly observes, the p-values associated with hypothesis tests for ZLA will depend on the number of tokens, and even more on the number of types, in the sample. Even a nearly perfect concordance between type duration and frequency of use can be marginally significant or nonsignificant if the number of types is small, and even a very weak concordance can be strongly significant if the number of types is large. In contrast, $\bar{\tau}$ is not sensitive to sample size. If we subsample from a large dataset, the expected value of $\bar{\tau}$ in the subsamples will be the same as $\bar{\tau}$ in the full dataset. This makes $\bar{\tau}$ useful for comparing the strength of ZLA among samples with different numbers of types. We now discuss this in lines 594-603.

Of course, the reviewer is correct that the statistical properties of communication systems might depend on the numbers of tokens or types in the systems or samples. If this is true, subsampling will not help, because systematic differences in the full dataset will be reflected in the subsamples. The best way to approach this is to choose the most relevant samples from each system. For birdsong, this is relatively easy. In some species birds can sing many different songs, but they do not usually sing many different *kinds of* songs. Even if they did, we could sample across those kinds of songs in proportion to the frequency with which they use them to obtain a reasonable measure of ZLA in each individual. In humans, this is less straightforward. Humans might produce everything from limericks to technical texts, the strength of ZLA might differ among those, and it is hard to know the “correct” frequency with which each type of text should be sampled. It is beyond the scope of this study to attempt to untangle how ZLA differs among text types in human languages. For this study, we used the Universal Declaration of Human Rights to represent written human language because i) it has been translated into many languages, and ii) it has been studied elsewhere (eg, Bentz and Ferrer-i-Cancho 2016) which facilitates comparison to past work.

In a comment below (p.17), the reviewer argues that we may have gone too far when we said that the differences in the strength of concordance we observed in birdsong and human language may indicate that the two systems follow different organising principles. This is a reasonable concern, and we have revised this statement and others like it throughout the text. We still observe that the strengths of concordance (ie, the concordances themselves, not simply the p-values associated with them) appear to be greater in human language than in birdsong. However, comparing the organising principles of the two systems will require new work, and we have removed claims about those here. Nonetheless, we suggest some hypotheses that might be tested in future work (lines 560-589).

p. 15 Sign of the correlations. "However, in 10 of 11 populations, the best estimates for the mean individual concordance were negative." The extensions of information theory developed by Ferrer-i-Cancho and collaborators, Ref 30, predict that the correlation should be negative (non-positive) in case of optimal coding. Namely, the prediction was successful in 10 out of 11 species! There is a theory that justifies/motivates authors' quest for ZLA in birds.

We agree. We have added a reference to this work in the introduction where we explain how ZLA is thought to arise (lines 71-73).

p. 17. "The negative concordance between phrase duration and frequency of use that we observed in birdsong is several times weaker than the negative correlation between word length and frequency of use that we measured in human languages. This may indicate that birdsong and human language follow different organising principles, and suggest limitations in the value of birdsong as a model for human language learning or processing."

These statements need to be revised. As explained above, the comparison between human language and bird song has not been made using the same modality and the same units and have not been matched by token size. The current finds do not question of the same principles apply. The authors have found that the predictions of the principle of compression hold for 10 out 11 species! If the samples of human language were down-sampled to match the number of "tokens" of bird phrases, would the comparison of humans versus birds be that shocking?

Please see our response to the comment above about controlling for the number of tokens in samples.

Table 1. I do not understand the meaning of the columns "phrase record^-1" and "phrase types record^-1"? What is -1? It does not seem the inverse, i.e. x^{-1}=1/x. Apologize my ignorance. It may be some standard I am not aware of.

It was meant to be the inverse. That is, “record^-1” is “1/record” is “per record.” This is a common notation in the ecology literature. But, the fact that the reviewer knew what it meant and was still confused shows that is not a good notation for a broad readership. We have replaced it with “per record” in all tables in the revised manuscript.

Statistical tests are one-sided or two-sided? In the legend of Table S2.1 the test are said to be one-sided. Same issue in Table S2.1

We apologise - this should have read one-tailed. But, even with this error corrected, we think this section of text was confusing, and perhaps inconsistent, in our original submission. In the revision, we explain that ZLAvian provides a one-tailed test for ZLA. That is, very low p-values indicate strong negative concordances, and p-values close to 1 indicate strong positive concordances (as opposed to no concordance at all, as they would in a two-tailed test; lines 456-461).

Table 1 does not show the number of individual birds for each species. It is actually shocking that after rounding to leave one decimal, the column "phrases record", that is the average number of phrases, ends up showing integer numbers except in a few cases. I may be missing something.

Each record is one bird, but we did not explain that in the original submission. We presented 3 significant figures for “phrases per record,” but we probably should have presented each value rounded to one decimal place as the reviewer suggests. We have corrected both errors in all tables in the revised manuscript.

Appendix 1. The authors present stochastic models that assume an exponential rank distribution for notes. Is this choice supported by the authors’ data or by previous research on bird song? If not supported, the question is to what extend the conclusions of that Appendix depend on that assumption. That looks easy to check for the authors as they performed a note level analysis in Appendix 2.

The assumption that note frequencies of use are exponentially distributed was a heuristic, but in retrospect it was a bad one. In practice, we know how frequencies of use are distributed, because it has been studied: they follow a Zipf-Mandelbrot law. In the revision, we have repeated the analysis assuming a Zipf-Mandelbrot distribution. We also present the original analysis to show that results are qualitatively similar either way (Supporting Information S3).

Consider also the work on duration of linguistic units other than "words" by Torre and colleagues, e.g. phonemes.

We thank the reviewer and now mention this work in the introduction (lines 75-76).

Appendix 2. p. 30 "and if ZLA operates in these populations it operates less strongly than in human languages." ZLA is just a statistical pattern derived by induction from data. ZLA does not operate, compression does. Of course, one can argue that there alternative mechanisms to the action of the principle compression (minimization of mean durations).

We have corrected this error here and throughout. (See our response to the similar comment above.)

Appendix 3. Fig. S3.1 shows plots in double logarithmic scale but the logarithmic scale does not look a very professional one. It seems that the authors have taken logs on x and y and then supplied log(x) and log(y) to the plotting tools. A professional plot in double logarithmic scale is obtained by supplying x and y to the plot function but then asking the scale to be logarithmic on both axes. In the authors plots, it is very difficult to know the true value of x and y (the authors do not indicate the base of the logarithm hence is log(x)=-4, the x could be 10^{-4}, e^{-4},...).

This is an excellent point. We have reformatted the axes in these figures (Figure 1 in the revised manuscript) to make them more intuitive and useful to readers. These figures were produced by the ZLAvian package, and we have updated ZLA to use the new formatting, and also to give users more control over how figure axes are displayed.
