# Supplementary material for: Does Zipf’s law of abbreviation shape birdsong?
Source: PLoS Comput Biol. 2025 Aug 13;21(8):e1013228. doi: 10.1371/journal.pcbi.1013228 (PMC12349147; doi:10.1371/journal.pcbi.1013228)
Supplement: Attachment — Submitted filename: responses to reviewers.docx [file pcbi.1013228.s007.docx]

#### Prof Natalia Komarova

#### Section Editor

*PLoS Computational Biology*

12 April 2025

Dear Prof Komarova,

My colleagues and I are grateful to you and the two anonymous reviewers for your comments on our revised manuscript. In particular, reviewer #1 raised two issues that they mentioned in the first round of review, but with clarifications and additional suggestions. We have carefully considered the reviewer’s advice and made several substantive changes in response, including adding two new analyses to the manuscript. We explain these in detail in our responses to the reviewers below. Reviewer #2 referred us to a relevant paper that was published while our manuscript was in review, and we now refer to this work in our introduction. We believe these revisions make our paper clearer and more useful to readers, and we are grateful for the advice.

Our resubmission includes two copies of the manuscript – a copy with changes tracked so you and the reviewers can easily see where we have made revisions, and a clean copy that may be easier to read. We have appended our responses to the reviewers’ comments below, with the reviewers’ comments in black and our responses in green. All line numbers in our responses refer to lines in the document with tracked changes showing.

We thank you again for your consideration.

Sincerely,

R. Tucker Gilman

Senior Lecturer in Environmental Research

Department of Earth and Environmental Science

University of Manchester

tucker.gilman@manchester.ac.uk

**Reviewers' comments:**

Reviewer #1: Although the authors have clearly put appreciable effort into revising their manuscript in light of the issues put forward by myself and the other reviewers, some of the main issues that I brought up in the last review have not been resolved in any substantive way. I am particularly concerned about the way that the results remain to be reported. For example, in the abstract, they still say that “we found weak trends consistent with Zipf’s law of abbreviation in 10 of the 11 populations we studied“ presumably referring to their observation of non-significant concordances observed in these populations. In combination with setting alpha at 0.1 in the previous revision, I am concerned that not reporting statistics in a principled way is a trend in this work, which is particularly important given that the main contribution of this work is in developing a statistical test. With that in mind I still believe that this work is a valuable contribution, and I ask that in a future version of this paper the authors will be more careful about ensuring that the claims they make align with their results.

We are pleased that the reviewer recognised our revisions as improvements and believes that the work is a valuable contribution. We address the reviewer’s concerns about the reporting of results in response to their specific points below.

Specific points:

The new analyses the authors made in reporting the strength of concordance needed to observe SLA given a dataset size is an important improvement in the current revision and I appreciate the authors adding this in.

Thank you. We are grateful for the suggestion in your first review.

As the authors note in their reply to my first review, this computed ‘maximum significant concordance’ is dependent on 3 factors: individual repertoire size, population repertoire size, and how similar repertoires are among birds. It would be valuable to report these numbers for some representative samples. For example, assuming a species had a 10%, 50% or 100% overlap in repertoires, you could plot a heatmap of the concordance needed for a range of individual and population repertoire sizes. Or similarity, holding the concordance at human level, you could plot what the required repertoire sizes would need to be. The point being - you don’t need to exhaustively sample these variables to give the reader a good sense of what sort of dataset they would need to collect from their species to determine whether ZLA is present.

We appreciate the reviewer’s suggestion. We agree that understanding the repertoire size needed to infer statistically significant ZLA in a population is valuable, and we focused much of our revision on this question. We found it to be even more complex than we initially expected.

The strength of concordance needed to infer statistically significant ZLA in a population depends not only on the individual and population repertoire sizes and on how note types are shared among birds, but also on the how much the frequencies of use vary among birds, and even on how much variability there is in the durations of notes that individual birds produce. In nature, these properties of birdsong vary among populations. They will also vary in simulated birdsong, but perhaps not in the same way they vary in nature. Thus, if we study the relationship between repertoire size and detection thresholds in simulations, our results may not tell us about birdsong as it appears in nature, but instead about birdsong as it exists in our simulations, and this will reflect the assumptions of the generating model rather than the true empirical patterns. Readers will be interested in understanding the relationship between repertoire size and detection thresholds in real populations, and therefore we believe the best way to study this relationship is by studying it in real populations. To this end, we have added a new analysis in which we regress the repertoire sizes in our study populations on the detection thresholds that we were able to achieve in those populations. We use this analysis to predict the repertoire size one would need to infer statistical significance for a given strength of ZLA. We also compute and present the 95% confidence intervals for the expectation of the required repertoire size. We believe this aligns with the reviewer’s goal of helping readers understand necessary sample sizes. Please see lines in methods 460-466, lines 491-495 in results, lines 528-538 in discussion, and Figure 3 for edits resulting from this suggestion.

To be clear, our goal in focusing on the empirical data is not to avoid doing the simulations that the reviewer suggested. In fact, we have done those simulations, and the results helped us decide to study the real data instead. Specifically, we used the birdsong model from Appendix S3 to generate simulated datasets with different population and individual repertoire sizes, numbers of birds, and note sharing patterns among birds. We controlled these attributes of the data in different ways, in particular:

1. *Increasing or decreasing the number of birds in the simulated data*. We recorded larger repertoires in simulations where we sampled more birds.
2. *Changing the distribution of note frequencies by changing the alpha parameter in the Zipf-Mandelbrot note frequency distribution*. When alpha is large, common note types are very common and rare note types are very rare. When alpha is small, note type frequencies are more similar. Repertoire sizes were smaller when note types were unevenly distributed because some rare note types were never encountered.
3. *Changing the innovation rates* (ie, the rate at which sons produce notes that are not learned from their father’s repertoires). Higher innovation rates resulted in repertoires with more note types and less note sharing.
4. *Changing the number of notes we sampled from each bird.* Samples with more total notes tended to have more note types.

In figure R1, we show that the relationship between the repertoire size and the detection threshold depends on how we control the repertoire size. Put another way, the repertoire size we need to infer statistically significant ZLA depends on the other parameters in the model.

Figure R1: The relationship between repertoire size and detection threshold as the population repertoire size changes with the number of birds sampled (blue), the frequency distribution of notes (black), the innovation rate (red), or the number of notes recorded per bird (green). All other parameters are held constant. Note the different lines of best fit (from generalised additive models) when repertoire size is controlled in different ways.

How the detection threshold depends on the repertoire size is also influenced by interactions between the parameters that control repertoire size. For example, in figure R2 we plot the detection threshold as a function of the population repertoire size when population repertoire size changes with the number of birds in the sample, and when the background innovation rate (the proportion of notes that sons do *not* learn from their fathers) in the model is 0.5, 0.1, or 0.01.

Figure R2: The relationship between repertoire size and detection threshold as the population repertoire size changes with the number of birds sampled when the background innovation rate is 0.5 (black), 0.1 (red), or 0.01 (green). The repertoire sizes needed to achieve a particular detection threshold differ by up to an order of magnitude depending on what we assume about the innovation rate.

We can see from figure R2 that the repertoire size needed to achieve a particular detection threshold can differ by an order of magnitude depending on the parameters assigned in the model that simulates the songs. Thus, predictions we make about required repertoire sizes based on simulations will be sensitive to assumptions about how songs are organised and passed among individuals and generations. We do not have reliable parameter estimates from nature to underpin our simulations. In fact, we do not even have a reliable simulation model – our simulation model is plausible, but other equally plausible models are possible. So, we cannot be confident that our predictions will be accurate for real systems.

Among the methods we used for manipulating repertoire size in our models, controlling the number of birds has the advantage that we can apply it to real populations. To do this, we took the three populations in our study that had the most birds (California thrashers, 89; black-headed grosbeaks, 83; Cassin’s vireos, 296) and we created samples with smaller repertoire sizes by subsampling from the birds in the full population (figure R3). Thus, the points in figure R3 represent samples that really might have been obtained from nature if the original researchers had sampled less intensively.

Figure R3: The relationship between repertoire size and detection threshold in subsamples from study populations of 83 black-headed grosbeaks (black), 89 California thrashers (red), and 296 Cassin’s vireo’s (green). The dotted horizontal line is the expected strength of ZLA in the populations we studied. The repertoire size needed to detect this strength of ZLA differs by more than a factor of two in these three populations.

Figure R3 shows that the relationship between repertoire size and detection threshold is likely to vary greatly even among real populations.

The figures above show that the relationship between repertoire size and detection threshold can depend on assumptions about how songs are organised and learned. However, perhaps there is a set of easily measured properties of simulated samples that, when taken together, accurately predict the repertoire size required to infer statistical significance in a population with a particular strength of ZLA. That is, maybe the generating model does not matter if we can measure the right properties of the simulated systems. If this is true, then a model fitted to the simulated data might also predict required repertoire sizes for real populations.

To test this, we regressed the repertoire sizes in our simulated birdsong samples on the detection thresholds, the number of birds, the mean repertoire size of each bird, and the mean number of notes sampled per bird in those simulated samples. We used the fitted model to predict the repertoire sizes we would need to achieve the observed detection thresholds in the real data (ie, from Bird-DB). We computed the difference between the predictions and the observed repertoire sizes in the real data. Then, we used our model of repertoire size as a function of detection threshold in the real data (ie, the linear model in figure 3 in the body of the paper) to predict the repertoire size we would need to achieve each real detection threshold, and we computed the deviations in this strictly empirical model. We compared the model based on simulations to the model based only empirical data with an F-test of the errors in their predictions. The model based on multiple predictors in the simulated data was not a better predictor of the observed repertoire sizes than the model based only on the detection threshold in the empirical data (F-test, df=10, p=0.5865).

For all of these reasons, we believe that the best approach to estimating the repertoire size needed to achieve statistical significance for a given strength of ZLA in a population is to estimate it from the linear model of the empirical data. This is the approach we have taken in our revision.

Finally, we reiterate an important idea from the body of the paper: *estimates of the strength of ZLA in a population are valuable even if the observed concordances are not statistically significant*. Note and phrase repertoires in birdsong are often small, and it may not be possible to infer statistically significant evidence of ZLA in populations with small repertoires. In such cases, we will *never* be able to say with confidence that there is ZLA in that population. Nonetheless, if we find negative concordances across many populations, we may be able to infer that the expected concordance in birdsong within species and populations is negative, even if the concordance within each population is not by itself significantly negative. We discuss this in lines 528-538 of the discussion, and in response to one of the reviewer’s comments below.

I can see documentation for your code on CRAN. All I see in the github repo is a readme and a csv file. Please upload your code and put a minimal script (or notebook) that reads that CSV, runs your analysis, and reports the results on github.

We apologise for this. The github repository was created only to share files among the authors. Our annotated code and data are publicly available on FigShare (doi:10.48420/24586791). The link was in the data availability statement we included with the submission, but we did not realise that this would not be shared with reviewers. We have corrected the error.

I would strongly suggest the authors do not claim that they observe “weak trends”, which here appears to mean the same thing as “trending towards significance”. See [https://doi.org/10.1136/bmj.g2215 [doi.org]](https://urldefense.com/v3/__https:/doi.org/10.1136/bmj.g2215__;!!PDiH4ENfjr2_Jw!Fns3vk1WB6r1RgBbUu2WJ-zGERoMZ_z3k8oAkBfMaK7cE7OTgBsH_FTEr-hsBf9CJiDJe5bnXqI1os0aV_4vfzCzr0w$) for a discussion as to why. If the authors of this work are not clear in what the output of their method means, how are the researchers who are using their tool supposed to understand the results on their own data? If the authors want to “give readers enough information to decide how much credence to put in our results“ by avoiding significance testing, they should be more principled in reporting the level of evidence e.g. by using established information theoretic methods (See Burnham, Anderson, and Huyvaert, 2011;
[https://link.springer.com/article/10.1007/s00265-010-1029-6 [link.springer.com]](https://urldefense.com/v3/__https:/link.springer.com/article/10.1007/s00265-010-1029-6__;!!PDiH4ENfjr2_Jw!Fns3vk1WB6r1RgBbUu2WJ-zGERoMZ_z3k8oAkBfMaK7cE7OTgBsH_FTEr-hsBf9CJiDJe5bnXqI1os0aV_4v1xSl0w8$))

The author states “it may be that the only way to assess ZLA in birdsong generally is to study many populations and ask if weak trends in the direction of ZLA are more common than we would expect by chance. We explain this in lines 120-130 of the introduction” but also that “many labs and researchers have datasets from a range of species that could be analysed using our method.
Aggregating and analysing that data is beyond the scope of this study, but we
believe that the ZLAvian package will make such analyses easy.”

The reviewer makes an important point. Many bird populations have small note or phrase repertoires, and it may be impossible to find statistically significant evidence for ZLA in birdsong in such populations. Therefore, one may need to synthesise evidence from many populations to understand whether ZLA is present in birdsong in general. We argue this in the introduction to the ms (lines 133-142), so it was remiss of us not to follow through and do it. We have corrected that in this revision. Instead of simply reporting that concordances were negative in 10 of 11 populations and leaving readers to decide (or perhaps wonder) what that means, we now formally analyse the set of concordances we observed. We cannot use the Burnham and Anderson approach that the reviewer suggests. That approach is designed to synthesise the results of multiple models applied to the same dataset, and our goal is to synthesise the results of the same method applied to multiple datasets. Mixed effects models, which are common in metanalysis, are well-suited to this task. We use a mixed effects model to ask whether the mean expected concordance in birdsong within a population and species is significantly less than zero. We supplement this with a Bayesian hierarchical model when we compare ZLA in birdsong to ZLA in written human languages – the Bayesian approach allows us to account for unequal variance spread among an unequal number of levels in the birdsong and human language data. We describe the additional methods in lines 423-431 and 439-444 and present the results in lines 486-490 and in the updated figure 2. Because we now have a formal test for the hypothesis that the expected concordance in birdsong within species and populations is negative, we no longer rely on the observation that 10 of 11 observed concordances were negative. We still report this (because it is still true!), but we have removed it from the abstract and been clearer in the discussion about how it can and cannot be interpreted (lines 497-550).

This said, we disagree with the reviewer’s contention that our observation of nonsignificant trends is similar to “trending towards significance.” That would be true if we argued that the negative concordances we observed in any particular bird population would become significant if we had larger repertoires to study. We make no such claim. The observed concordances within populations and the p-values that describe them are what they are, and nothing more. Taken individually, none of them is strong evidence for ZLA. Nonetheless, it is certainly true that 10 of the 11 observed concordances are negative. One could use a binomial test to find the probability that we would see 10 negative concordances in 11 populations if positive and negative concordances were equally common in birds, as we would expect under the null. The answer is p = 0.0059. The reason we did not do this in the ms is that there would be a question of HARKing (sensu Hollenbeck and Wright, <https://doi.org/10.1177/0149206316679487>) in the choice of the binomial test. Furthermore, we are interested in more than the sign of the expected concordance – we are interested in its magnitude – and a binomial sign test does not estimate this. The mixed model and Bayesian hierarchical model both allow us to estimate the magnitude of concordance, and thus are more appropriate and more useful to readers.

All quibbling about “trending to significance aside,” we are grateful to the reviewer for persisting with this idea. We think the additional analysis greatly improves the manuscript.

It would be valuable, at least in the discussion, to spend some time trying to estimate what effort future researchers would need to make to perform an analysis powerful enough to properly test for ZLA in birdsong. For example, which species produce a large enough repertoire to study ZLA?

We agree. We have added an analysis to address this (figure 3), and we discuss this in lines 491-495 and 528-538. See also our extended response above.

Are there corrections for multiple tests being performed across species? Here there are 11 groups tested, and 2 are significant. I want to make sure this result is performing corrections to correct for multiple tests.

We make no claims in the paper about the significance of ZLA in individual populations, so there is nothing to correct. However, we share the reviewer’s concern that readers should not overinterpret the p-values associated with ZLA in the individual bird populations. We now clarify the interpretation of the p-values for the individual populations in the abstract (lines 31-33) and discussion (lines 497-506).

Reviewer #2: Thank you for taking my and the other reviewers' recommendations on board. I think the motivation could still be written in a more convincing way, but it isn't such a big issue that it should further delay publication. My other concerns have been addressed in the revised ms which is much improved, and I'm happy to recommend acceptance. As a small addition, I recommend including the following paper (just published last week) which shows Zipfian distribution in humpback whale songs as a citation: Inbal Arnon et al. (2025). Whale song shows language-like statistical structure. Science 387,649-653. DOI:10.1126/science.adq7055.

Thank you. We have now included this paper, and a similar paper by Youngblood that appeared in the same week ([DOI: 10.1126/sciadv.ads6014](https://doi.org/10.1126/sciadv.ads6014)) (line 89).
